# Implications of Domestication in *Theobroma cacao* L. Seed-Borne Microbial Endophytes Diversity

**DOI:** 10.1007/s00248-024-02409-9

**Published:** 2024-08-28

**Authors:** Deisy Lisseth Toloza-Moreno, Roxana Yockteng, José Ives Pérez-Zuñiga, Cristian Salinas-Castillo, Alejandro Caro-Quintero

**Affiliations:** 1 Centro de Investigación Tibaitatá, Corporación Colombiana de Investigación Agropecuaria (AGROSAVIA), Km 14 Vía Mosquera, Cundinamarca, Colombia; 2 Institut de Systématique, Evolution, Biodiversité-UMR-CNRS 7205, Muséum National d’Histoire Naturelle, Paris, France; 3 Centro de Investigación Palmira, Corporación Colombiana de Investigación Agropecuaria (AGROSAVIA), Sede Popayán, Popayán, Cauca Colombia; 4 Departamento de Biología, Facultad de Ciencias, Max Planck Tandem Group in Holobionts, Universidad Nacional de Colombia, Bogotá, Colombia

**Keywords:** Seed-borne microbial endophytes, *Theobroma cacao*, ACC deaminase activity, Indole acetic acid

## Abstract

The study of plant–microbe interactions is a rapidly growing research field, with increasing attention to the role of seed-borne microbial endophytes in protecting the plant during its development from abiotic and biotic stresses. Recent evidence suggests that seed microbiota is crucial in establishing the plant microbial community, affecting its composition and structure, and influencing plant physiology and ecology. For *Theobroma cacao* L., the diversity and composition of vertically transmitted microbes have yet to be addressed in detail. We explored the composition and diversity of seed-borne endophytes in cacao pods of commercial genotypes (ICS95, IMC67), recently liberated genotypes from AGROSAVIA (TCS01, TCS19), and landraces from Tumaco (Colombia) (AC9, ROS1, ROS2), to evaluate microbial vertical transmission and establishment in various tissues during plant development. We observed a higher abundance of *Pseudomonas* and *Pantoea* genera in the landraces and AGROSAVIA genotypes, while the commercial genotypes presented a higher number of bacteria species but in low abundance. In addition, all the genotypes and plant tissues showed a high percentage of fungi of the genus *Penicillium*. These results indicate that domestication in cacao has increased bacterial endophyte diversity but has reduced their abundance. We isolated some of these seed-borne endophytes to evaluate their potential as growth promoters and found that *Bacillus*, *Pantoea*, and *Pseudomonas* strains presented high production of indole acetic acid and ACC deaminase activity. Our results suggest that cacao domestication could lead to the loss of essential bacteria for seedling establishment and development. This study improves our understanding of the relationship and interaction between perennial plants and seed-borne microbiota.

**Supplementary Information:**

The online version contains supplementary material available at 10.1007/s00248-024-02409-9.

## Introduction

Beneficial microorganisms colonize most plants on the planet, and the composition of these microbial communities is influenced by multiple factors, which vary according to the host species, tissues, plant phenology, geographic location, and environmental conditions [1]. In many cases, these microbial communities play important roles that affect plant health, for example, protection against pathogens, nutrient fixation and translocation, drought resistance, and resistance to herbivory [2]. This evidence shows that the relationship between microorganisms and plants is fundamental in plant biology, and it is essential to understand, for instance, seed germination, plant establishment, and adaptation to stressful conditions [3, 4].

Few studies have focused on determining the effects that influence the formation of a microbial community in a host and how these factors might modify the host-microbe interaction [5]. Among these effects, some can occur by changes in the environmental conditions originated by microorganisms already established in a host, which affect the establishment of new species in the community [6, 7]. This phenomenon is known as priority effects [8]. Within the seed, these priority effects may occur due to the presence of primary symbionts that may affect the ability to form secondary symbiotic relationships in maturing hosts [9] and sometimes exclude other microorganisms due to competition [10]. Therefore, this community of microorganisms might have a role in the composition, assembly, establishment, and structure of the mature plant's microbial community and, consequently, in the plant physiology and ecology [4, 7, 11, 12].

The microbial populations can colonize the external plant surfaces (epiphytic microbiota) or live in the internal plant tissues (endophytic microbiota). Plants would acquire endophytic microorganisms horizontally from the soil and the surroundings or through vertical transmission (seed-borne) [4, 13]. Even though many of these microorganisms' roles are still unknown, the studies suggest that they are not pathogenic and are often beneficial for the host plant [6, 11]. In seeds, fewer studies have evaluated the diversity of endophytes, the mechanisms that structure microbial communities within these [14], and their vertical transmission or establishment in different tissues during plant development [13]. Bacterial endophytes can inhabit various parts of the seed, such as the embryo [15], and after germination, they can colonize the developing tissues [4, 16], such as stems and leaves, and eventually the entire plant endosphere [15]. Certain endophytes can also colonize flowers and seeds and may be transmitted vertically to the next generation [14, 17].

In addition, seed-borne microbial endophytes are particularly interesting as they enhance seed germination and plant growth promotion, so they are important in improving crop productivity and resilience [18–20]. Thus, they can be used as bioproducts in phytoremediation and sustainable agriculture [21]. Also, they might evolve mechanisms that protect the germinating seeds and seedlings from foraging, drought, and fungal seed-borne and soil pathogens [7]. One of the most fascinating interactions between these microorganisms and their host plants is the physiological control of plant growth by bacteria, in which bacteria produce plant hormones or hormone-related signaling molecules that modulate the plant stress response [22]. From these, indole-3-acetic acid (IAA) is an essential hormone in plant-bacterial interactions [12], which improves plant growth under stress conditions [23]. IAA is a plant auxin involved in various plant physiological processes, such as regulation of plant development, induction of plant defense systems, and formation of lateral and adventitious roots [24]. The endophytic bacteria use the tryptophan secreted by plants in the rhizosphere as a precursor for IAA biosynthesis [24, 25]. They also produce the enzyme 1-aminocyclopropane-1-carboxylate (ACC) deaminase that hydrolyzes ACC, an ethylene precursor regulating its production and metabolism. Ethylene controls the response of plants to abiotic and biotic stresses inhibiting root development [12]. ACC hydrolysis decreases plant stress by improving plant growth under stress conditions and helps to produce deep roots for better plant water absorption [25].

Recent data on multiple cultivated species indicate that the seed microbiota would constitute most cultivated plants' microbial populations [17]. The study of seed-borne endophytes has focused on short-cycle crops (*e.g.,* maize, soybean, rice) where the effect of endophytes on establishment, growth, and resistance to stresses has been evaluated [26–29]. For tropical perennial plants, studies on the association and interaction of plants and seed-borne microbiota are still incipient. A study in wild perennial plants showed that the composition of microbiota changes during the lifetime [30]. For instance, a study in *Quercus robur* reported that under controlled conditions, a significant fraction of the seed microbiome was transferred to the root tissue of developing seedlings [31].

Microbial communities also have an essential role in another perennial species as *Theobroma cacao* L. (cacao), which produces the main ingredient of chocolate. It has been reported that these microbial communities affect the quality of the fermented cacao bean and, eventually, the quality of chocolate, and thus, several studies have focused on determining their importance in the fermentation process [32–35]. Interestingly, some of the observed microbes during the early fermentation times belong to seed endophytes recurrently found in other plants [17], such as *Tatumella* and *Pantoea* bacteria [33, 36]. Studies of microbial endophytes associated with cacao plant tissues and their role are scarce. For instance, Simarmata et al*.* [37] identified the endophytic bacteria *Brevibacillus brevis* and *Pantoea* sp. as cacao growth promoters, and Hanada et al*.* [38] found that endophytic fungi of the genera *Trichoderma*, *Pestalotiopsis*, *Curvularia*, *Tolypocladium,* and *Fusarium* showed antagonistic capacity against *Phytophthora palmivora*. Further studies are needed to determine endophytes' acquisition and transmission mechanisms and their possible functions in *T. cacao*.

Here, we present the first study of the composition of the seed-borne endophytic microbial community of *T. cacao*. We germinated seeds under sterile conditions to assess the diversity of the seed-borne microbiota and their establishment in seedling tissues. Bacteria and fungi diversity was identified in different seedling tissues by sequencing molecular markers (16S rRNA and the Internal Transcribed Spacer ITS-5.8S). Additionally, we isolated bacteria and evaluated their plant growth-promoting capabilities. We analyzed the variability of these communities and the possible effect of domestication by comparing widely used commercial cacao genotypes, AGROSAVIA genotypes, and landraces from Tumaco (Nariño, Colombia). Exploring the diversity of seed-borne endophytes in cacao opens the door not only for elucidating the role of vertical transmission in plant success but also for identifying and modifying the communities involved in cacao bean fermentation and chocolate quality.

## Materials and Methods

### Germination and Emergence of Cacao Seedlings

Pods of seven genotypes of *T. cacao* were harvested at the AGROSAVIA's El Mira Research Center and cacao-producing farms in Tumaco, Nariño (Colombia), obtained from the commercial genotypes (IMC67, ICS95), recently liberated genotypes from AGROSAVIA (TCS01, TCS19), and landraces from Tumaco (AC9, ROS1, ROS2). Commercial genotypes were collected in 1930 and have been used in breeding programs since 1960; recently liberated genotypes were introduced to farmers in 2014, and landraces have not undergone any breeding improvement and, therefore, have not been massively propagated.

Disinfection of cacao pods and seeds was performed using the methodology described by Falcäo et al*.* [39] with modifications. Cacao pods were washed with soap, disinfected with 2% iodine solution and 70% ethanol, and rinsed with sterile distilled water (sdw). We opened the pods to extract the seeds, manually removed the mucilage using sterile river sand, and rinsed with sdw. Successive washes were performed under constant agitation using 70% ethanol for 2 min, sdw for 2 min, 2.5% sodium hypochlorite for 7 min, and sdw for 2 min. Final disinfection was done with 2.5% sodium hypochlorite for 7 min in a laminar flow cabinet, and seeds were rinsed three times with sdw for 2 min.

Seeds from each cacao genotype were germinated in a humid chamber at room temperature between five and nine days until radicles reached a length of 1.5—2.0 cm. Thirty-six germinated seeds of each cacao genotype were transferred individually to a sealed 500 mL sterile glass vial with wet sterile river sand and placed in a growth room at 26 ± 2 °C and 60% relative humidity. We recorded the emergence and development of seedlings for five weeks, making weekly observations. At the fifth week, we selected ten seedlings per genotype with stems, leaves, and leaflets properly developed. We extracted them from the substrate in a laminar flow cabinet and stored them at -80 °C for further use.

### DNA Extraction and Library Preparation

Root, stem, leaf, and cotyledon tissues from the stored seedlings (ten per genotype) were macerated separately in liquid nitrogen. DNA extraction was performed using the DNeasy® Plant Mini Kit (QIAGEN). The quality and concentration of the DNA were verified by visualization in 1% agarose gels stained with SYBR Safe (Invitrogen Carlsbad, CA, USA) and by quantification in a NanoDrop™ 1000 UV spectrophotometer (NanoDrop Technologies, Wilmington, DE, United States), respectively.

The 16S rRNA (515F – 806R) [40] and ITS-5.8S (ITS3_KY02 – ITS4_KY03) [41] regions were amplified to characterize the endophytic bacterial and fungal communities associated with the plant tissues. DNA library preparations followed the protocol described in Caporaso et al*.* [40] and Caro-Quintero and Ochman [36]. In the first PCR, the primers used to amplify each region have an additional sequence at the 5' end that serves as a linker sequence for the indexing step and sequencing primer region. In the second PCR, indexes and the i5 and i7 Illumina primers were added to each amplicon [32]. The PCR products obtained in each amplification were purified with Agencourt AMPure XP Beads magnetic beads (Beckman Coulter, Brea, CA, USA) and verified by electrophoresis in 1.5% agarose gels stained with SYBR Safe.

For the first PCR, reaction volumes of 20 and 25 µL were used to amplify 16S rRNA and ITS-5.8S regions, respectively. Each reaction for 16S rRNA was composed of 0.4 µL Phire Hot Start II DNA Polymerase (Thermo Scientific), 4.0 µL Buffer 5X, 0.4 µL of dNTPs (10 mM), 0.5 µL of each primer (10 µM), 2.5 µL DNA, and 11.7 µL of ultrapure distilled water (udw) (Invitrogen Carlsbad, CA, USA). The PCR program consisted of an initial denaturation step at 98 °C for 60 s, followed by 35 cycles: denaturation at 98 °C for 30 s, annealing at 60 °C for 10 s, and extension at 72 °C for 15 s, and a final extension step of 72 °C for 120 s. For ITS-5.8S, each reaction was composed of 0.25 µL Phire Hot Start II DNA Polymerase, 5.0 µL buffer 5X, 0.5 µL dNTPs (10 mM), 1.0 µL of each primer (10 µM), 3.0 µL DNA, and 14.25 µL of udw. The PCR program consisted of an initial denaturation at 98 °C for 30 s, followed by 35 cycles: denaturation at 98 °C for 10 s, annealing at 55 °C for 30 s, and extension at 72 °C for 90 s, and a final extension step of 72 °C for 120 s.

For the second PCR, each region was amplified in a 15.0 µL reaction volume. Each reaction was composed of 0.3 µL Phire Hot Start II DNA Polymerase, 3.0 µL buffer 5X, 0.3 µL dNTPs (10 mM), 0.8 µL of each barcode, 3.5 µL of the amplicon of the first PCR, and 6.3 µL of udw. The PCR program consisted of an initial denaturation step at 98 °C for 30 s, followed by 17 cycles of denaturation: 98 °C for 0.05 s, annealing at 50 °C for 16S rRNA and 55 °C for ITS-5.8S for 30 s, and extension at 72 °C for 60 s, and a final extension of 72 °C for 60 s.

Finally, the purified DNA libraries were quantified in a NanoDrop 1000 Spectrophotometer, adjusted to the same concentration, and pooled in one sample. The quality and concentration of pools were verified by automatized electrophoresis in an Agilent 4200 TapeStation System (Agilent Technologies) and sequenced in an Illumina MiSeq System (Universidad del Bosque, Colombia).

### Estimation of the Diversity and Structure of the Microbial Community

We assessed the quality of the reads using FastQC v.0.11.5 [42]. We performed demultiplexing, pair-end assembly, and diversity analysis using QIIME2 [43]. Dada2 [44] was the algorithm used to denoise reads and generate Amplicon Sequence Variants (ASVs). Dada2 used the paired-end sequences as input with a truncation length of 200 bp for forward and 240 bp for reverse reads.

Taxonomic classification of bacteria ASVs was performed using the GreenGenes database [45], v-gg-13–8-99-nb [43, 44]. We compared the number of ASVs associated with mitochondria to the total bacteria ASVs per sample to establish the relative abundance of bacteria in the different genetic genotypes and tissues. The fold change ratio between the two was computed (log2(Bacteria/Mitochondria)) to normalize bacterial abundance per tissue. For the taxonomic classification of fungi, two approaches were used. First, ASVs were classified using the UNITE database (unite-ver8-taxonomy_99_10.05.2021_dev.fasta) [46]. Then, ASVs were searched using BLAST (blastn) against the Internal transcribed spacer region (ITS) from fungi type and reference material from the NCBI (National Center for Biotechnology Information) to determine the closest taxonomic group associated with each library. The ASVs were assigned to the taxonomic group with the best hit score. When multiple taxonomic groups had the best hit score, the ASVs were assigned to the broader consensus taxonomic group.

For the quantification of alpha and beta diversity, we removed the ASVs corresponding to chloroplast and mitochondria from the representative sequences as well as the feature tables using the program "QIIME taxa" with "filter-seqs" and "filter-table" options.

Rarefaction curves were estimated to determine the minimum read depth that reaches species saturation for most libraries. The 16S rRNA libraries were sub-sampled to the minimum depth of 300 reads, while ITS-5.8S libraries were sub-sampled at 900 reads. To estimate species richness (alpha diversity), we quantified the Shannon, Faith, and Phylogenetic Diversity (PD) indices. We conducted a pairwise Kruskal–Wallis test to assess the statistical differences between genotypes and tissues.

We determined the similarities and differences between samples (beta diversity) by calculating the weighted and unweighted UniFrac metrics [47]. We conducted a Principal Coordinate ordination Analysis (PCoA) using the UniFrac metrics distance matrix to visualize the ordination of samples and how variables, such as the genotype and tissue type might determine the observed clustering. A permutational multivariate analysis of variance, PERMANOVA [48], was performed using the unweighted and weighted UniFrac distance matrix to establish the significant differences between groups of samples.

### Isolation and Molecular Identification of Bacteria from Cacao Plant Tissues

We isolated bacteria from seedlings from all genotypes. We separately macerated each plant tissue (seed, root, stem, leaf, and cotyledon) in a laminar flow cabinet. One gram of each macerate was collected and suspended in 9 mL of 0.85% sterile saline solution. Serial dilutions were prepared and plated in triplicate on Luria Bertani and Nutritive Agar culture media. For seeds, 100 μL of 10^–2^ to 10^–5^ dilutions were plated, while for the other tissues, 100 μL of 10^–3^ to 10^–5^ dilutions were plated. Petri dishes were incubated at 30 °C for 24, 48, and 72 h. After incubation, macroscopic (color, elevation, appearance, consistency, edge of colony) and microscopic (Gram-positive or Gram-negative bacteria, cell shape, and arrangement) descriptions of isolated colonies were made, and the identified morphotypes were individualized to obtain pure cultures. From these plates, colony morphotypes were picked and DNA extraction was done using the ZR Fungal/Bacterial DNA MiniPrep™ Kit. The obtained DNA was quantified in NanoDrop™ 1000 UV spectrophotometer. The 16S rRNA region was amplified using the 27F (5'-AGAGTTTGATCCTGGCTCAG-3') and 1492R (5'-GGTTACCTTGTTACGACTT-3') primers. Each PCR reaction was composed of 0.4 µL Phire Hot Start II DNA Polymerase, 4.0 µL Buffer 5X, 0.4 µL dNTPs (10 mM), 1.2 µL of each primer (10 µM), 3.0 µL DNA, and 9.8 µL of ultrapure distilled water. The PCR program consisted of an initial denaturation step at 98 °C for 60 s, followed by 35 cycles: denaturation at 98 °C for 30 s, annealing at 60 °C for 10 s, and extension at 72 °C for 15 s, and a final extension step of 72 °C for 120 s. The PCR products were verified by electrophoresis in 1.5% agarose gels stained with SYBR Safe and then purified with Exo-CIP™ Rapid PCR Cleanup Kit (New England BioLabs) and sequenced by the Sanger method (Universidad Nacional de Colombia).

### Assessing the IAA Production and ACC Activity of Bacterial Isolates

The potential of each morphotype for plant growth promotion was evaluated quantifying their capability, in vitro, to produce Indole Acetic Acid (IAA) and 1-aminocyclopropane-1-carboxylic acid (ACC) deaminase activity.

First, to evaluate IAA production, an inoculum of each morphotype was suspended in 1 mL of 0.85% sterile saline, and the absorbance was measured at 600 nm and adjusted to an optical density of 0.2. Then, 100 µL of this suspension were inoculated by triplicate in 5 mL of a minimal medium M9 (composition g.L^−1^: 3 g KH_2_PO_4_, 1 g NH_4_Cl, 0.5 g NaCl, 0.25 g MgSO_4_, 3 g filtered casamino acids) supplemented with 2.5 mM tryptophan and incubated at 30 °C for 72 h with agitation at 180 rpm in the dark. Then, 1 mL of each culture was centrifuged at 14000 rpm for 5 min, and 100 µL of the supernatant was taken and added to a microplate with 100 µL of Salkowski's reagent [49]. The microplate was left in the dark with agitation at room temperature for 30 min. Finally, the absorbance was measured by triplicate at 530 nm and compared with a calibration curve of IAA in concentrations from 1 to 45 ppm (part per million) dissolved in ethanol. The strain *Azospirillum brasiliensis* SP7 was used as a positive control, as it is an ATCC reference strain known to produce IAA.

The ACC deaminase initial screening was done following the protocol reported previously by Habib et al*.* [50]. Then, the isolates' ability to produce ACC deaminase was evaluated using the M9 medium. This medium was prepared by mixing 20 mL of a sterile solution of salts (8.5 g Na_2_HPO_4_, 1.5 g KH_2_PO_4_, 0.25 g NaCl dissolved in 100 mL of double-distilled water) with sterile solutions of 0.2 mL 1 M MgSO_4_, 10 µL of 1 M CaCl_2_, 2 mL of 20% glucose p/v, and distilled water to complete a final volume of 100 mL. The ACC deaminase assay was conducted in a 96-well microplate, one isolate per row. We deposited 120 µL of M9 medium in each well. We added 15 µL of 0.1 M MgSO_4_ solution to the first four wells of each row and 15 µL of 3 mM ACC solution to the following four wells of each row. A volume of 15.0 µL of bacterial inoculum was added in wells two to four and six to eight. Wells one and five were used as blanks, and an additional 15 µL 1 M MgSO_4_ was added to have the same total volume in all wells. The microplates were incubated at 30 °C with continuous agitation of the medium, and the absorbance was measured at a 600 nm wavelength every two h for two days using a BioTek Synergy HTX Reader (Agilent Technologies). The ACC deaminase activity was positive if the growth curve of the isolates with ACC was higher than the observed with 0.1 M MgSO_4._ *Pseudomonas putida* UW4, with recognized ACC activity, was used as a positive control, while a mutant of *P. putida* UW4 *acdS* was used as a negative control. As a blank, a solution without nitrogen was prepared (0.1 M MgSO_4_).

To identify the isolates that produced ACC deaminase, we calculated the difference between the cell growth in the medium with ACC and in the medium with MgSO_4_. With this information, we clustered the isolates using the Manhattan distance and visualized the results as a heatmap using the package heatmap from R.

## Results

### Seedling Development

The different cacao genotypes' germination, emergence, and development were evaluated under in vitro conditions. Over 75.0% of all genotypes' seeds germinated in five to nine days. The germination percentage was higher for IMC67, AC9, and ROS2 with 97.2%, followed by TCS01, TCS19, and ROS1, with 96.9, 88.6, and 77.8%, respectively. ICS95 was the cacao genotype with the lowest germination percentage (75.0%). Meanwhile, seedlings' emergence was higher for TCS01, with 71.0%, followed by ROS2, and AC9, with 65.7 and 62.9%, respectively. Cacao genotypes with the lowest seedling emergence percentages were TCS19, IMC67, ROS1, and ICS95, with 51.6, 51.4, 42.9, and 40.7%, respectively (Table S1).

### Library Preparation and Sequencing for 16S rRNA and ITS-5.8S Regions

By sequencing 16S rRNA and ITS-5.8S regions, we examined the microbial community composition for the different cacao genotypes. We obtained successful amplifications in 84.8% of the samples. The success in library construction varied between sequenced regions, genotypes, and tissue. For 16S rRNA, we constructed libraries for 93.2% of the samples, while for ITS-5.8S, the success of generated libraries was for 76.4% of the samples (Fig. S1).

For the 16S rRNA region, 266 libraries were successfully sequenced, with an average sequence depth of 5257.18 (± 2891.29) reads per sample. After performing the quality control, 263 libraries were retained, with 69.6% of the original reads (Table S2). For the ITS-5.8S region, sequences for 221 samples were obtained with an average sequence depth of 5718.12 (± 3123.20) reads per sample. Only 0.6% of the reads were lost after performing quality control, allowing the retention of 99.4% of reads. The sequencing depth for all the libraries ranged between 285 and 15385 reads, with an average of 5686.68 (Table S3).

### Genotypic and Tissue-Specific Variations in Bacterial Abundance

Taxonomic classification allowed us to separate the fraction of reads belonging to bacterial 16S rRNA from those belonging to plant organelles (mitochondria and chloroplast). Most reads were classified as mitochondrial or chloroplast 16 s rRNA, suggesting that the abundance of bacteria within seeds was low compared to the number of bacteria in other tissues.

**Fig. 1:**
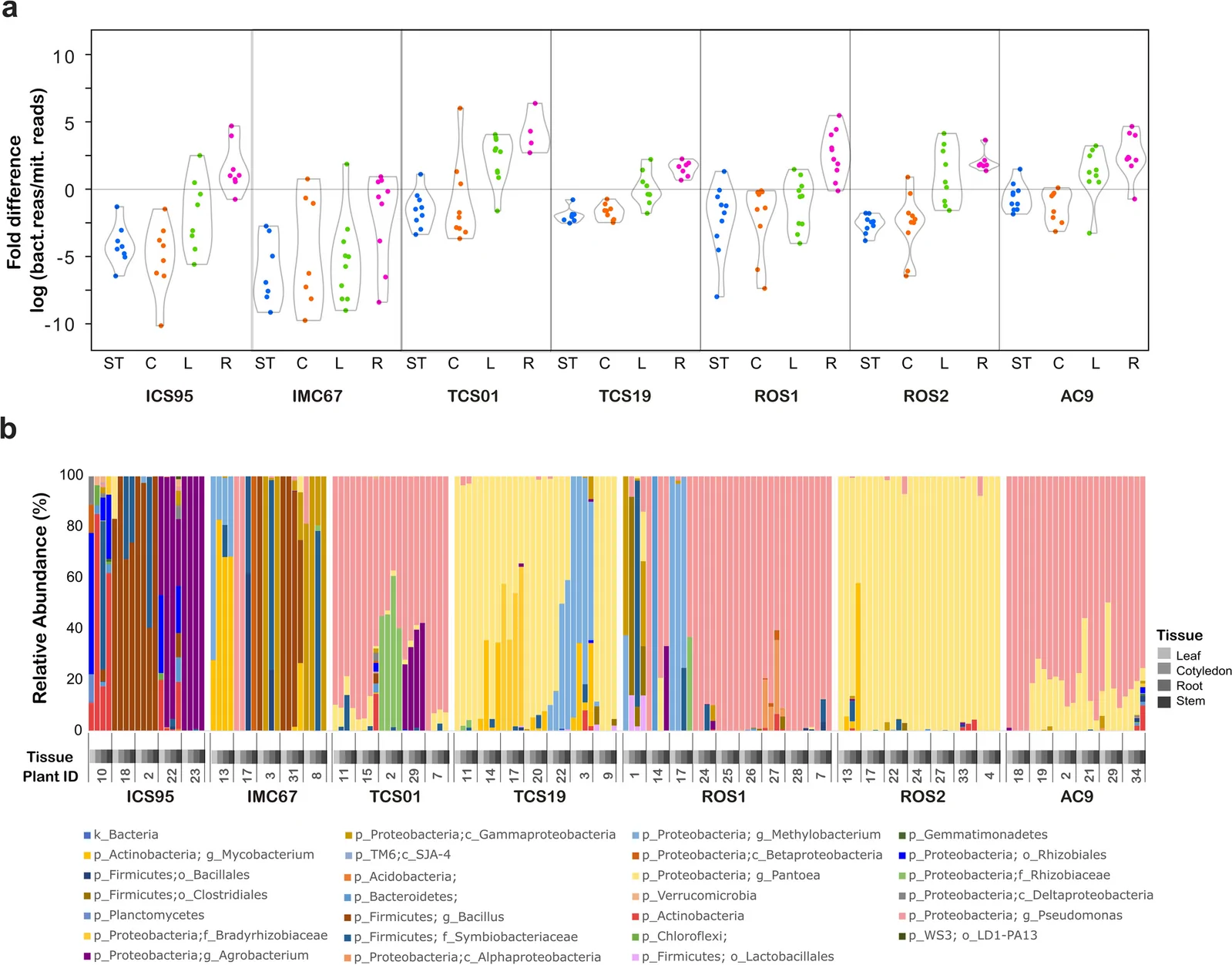
Relative abundance and taxonomic classification of seed-borne bacterial communities in different *T. cacao* genotypes and tissues. **a** The relative abundance of bacteria to mitochondria is presented per genotype and tissue (ST: Stem, C: Cotyledon, L: Leaf, R: Root). The fold difference represents the log2 of the ratio between bacterial and mitochondrial reads. Positive values represent tissues with a higher number of reads associated with bacteria than mitochondria, while negative values represent higher mitochondrial reads. **b** The corresponding taxonomic classification of bacterial reads is shown per genotype, seedling, and tissue. Seedlings replicates are identified by their assigned numbers. The different tissues, leaf, cotyledon, root, and stem are shown in a scale from light to dark gray. The genotypes were ordered left to right, from more to less domesticated. Only seedlings with data for the four analyzed tissues are shown

A closer examination of the data sets from seedlings showed that the relative abundance of reads classified as bacteria, chloroplast, and mitochondria was similar within tissues (*e.g.,* more similar within roots of all genotypes), showing that the relative abundance of bacteria is tissue dependent. To compare the taxonomic diversity patterns of bacteria between tissues and their relative abundance, we used the mitochondrial 16S rRNA as an internal marker. This marker allowed us to normalize the abundance of bacteria in each sample. To explore these differences, we calculated the fold change between the number of bacteria and plant mitochondria 16S rRNA reads and evaluated the relative abundance of bacteria in each plant tissue for each cacao genotype (Fig. 1).

The bacterial to mitochondrial proportion of 16S rRNA reads reveals tissue-specific patterns of bacterial abundance. Roots tissue had the highest abundance of bacteria reads compared to mitochondria, in the case of TCS01, bacterial reads in root tissues were 31.7 times higher than mitochondria, and similar tendencies were found in ROS1, AC9, ICS95, ROS2, and TCS19 genotypes with 11.3, 9.3, 6.7, 4.8 and 3.0 times more bacterial reads. Only IMC67 had a lower number of bacterial to mitochondrial reads with 0.87 times. The leaves tissue was the second with the highest bacterial abundance, in this case, TCS01, ROS2, ROS1 AC9, and TCS19 genotypes had a higher number of bacteria with 7.7, 4.7, 3.7, and 1.5 times the number of mitochondria while IC95 and IMC67 had lower abundance with 0.98 and 0.41 times. Cotyledon and stem tissues had a lower abundance of bacteria in all genotypes as shown by the negative values of the fold difference (Fig. 1a), exceptions were observed for three seedlings of the TCS01 genotype.

The fold difference analysis also shows differences among genotypes. The AC9, ROS2, ROS1, TCS01, and TCS19 genotypes had a higher average value of bacterial abundance, with 3.6, 2.5, 3.3, 12, and 1.3 times the number of bacterial to mitochondrial reads. This contrasts with the observed in the most domesticated genotypes IMC67 and ICS95 with average values of 0.37 and 0.96. In all evaluated tissues, the IMC67 genotypes had the lower overall bacterial abundance; for instance, tissues such as the IMC67 stem had almost 32 times more mitochondria than bacteria on average (a fivefold difference, Fig. 1a).

### Bacterial and Fungal Taxonomic Composition

Bacterial composition was evaluated after excluding 16S rRNA plant mitochondria and chloroplast sequences. We obtained 2,514,150 reads belonging to 240 libraries. These reads were classified into taxonomic groups using the GreenGenes database. Most of the 16S rRNA reads were assigned to the *Proteobacteria* (86.3%) and the *Firmicutes* phyla (11.3%, Fig. 1b). A few other reads were assigned to the phylum *Actinobacteria* (2.2%).

At a finer resolution, we found 87 groups at the genus level in all samples. Of these 87, only 61 had more than 1.0% abundance. These genera were not shared by all samples; for instance, only 33 were in more than one sample. The genus *Pseudomonas* was the most prevalent, being identified in 107 plant tissues of the 280 evaluated, followed by the genus *Pantoea*, which was found in 102 tissues. *Pseudomonas* was dominant in the tissues of AC9, ROS1, and TCS01 genotypes, while *Pantoea* was more prevalent in ROS2 and TCS19. The abundance of these two genera was negatively correlated (*r* = -0.47, *p* < 0.05), indicating possible ecological exclusion. In the case of ICS95 seedlings, *Agrobacterium* and *Bacillus* were predominant. In contrast, in IMC67, we did not observe the prevalence of one specific bacteria genus. Instead, we detected the presence of multiple genera and families, such as *Methylobacterium, Mycobacteriaceae*, *Symbiobacteriaceae*, and *Bradyrhizobiaceae* (Fig. 1b).

Some differences were observed between seedlings from the same genotype. For instance, seedling 34 of the AC9 genotype presented a greater diversity of bacteria than the other replicates. In ROS1, three of the seedlings evaluated presented taxonomic groups that were more prevalent than those found in the other replicates. For seedling 17, the genus *Methylobacterium* predominated, except in the root where *Bacillus* and *Pseudomonas* genera were detected. In seedling 14, only the phylum *Bacteroidetes* was presented in the stem, and in seedling 27, a dominant group belonging to the class *Alphaproteobacteria* was observed (Fig. 1b).

**Fig. 2:**
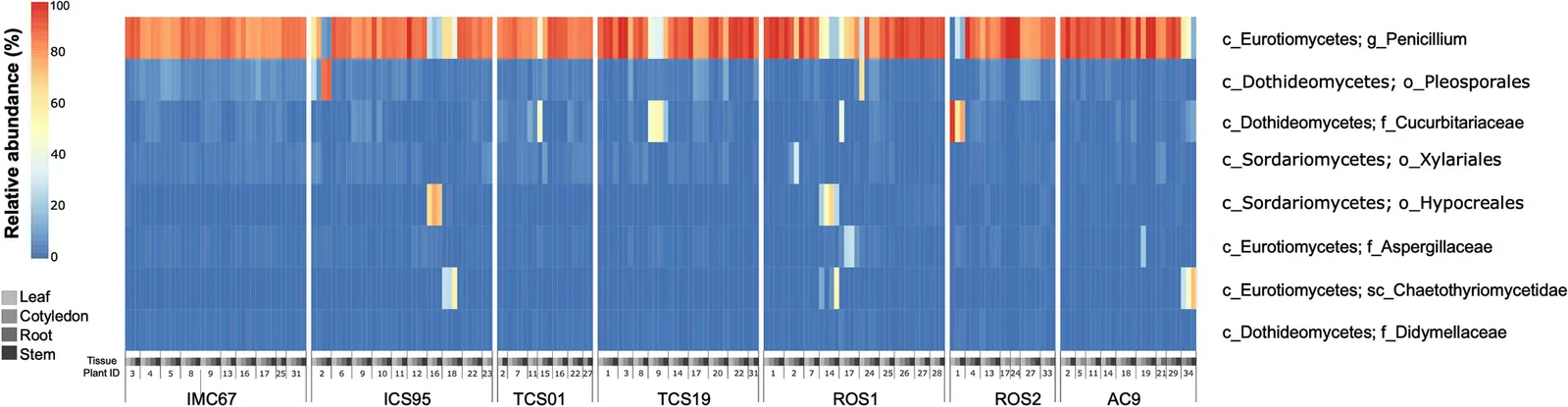
Relative abundance and taxonomic classification of dominant fungi communities in *T. cacao* commercial genotypes (IMC67 and ICS95), recently liberated genotypes from AGROSAVIA (TCS01 and TCS19), and landraces (ROS1, ROS2, and AC9). The lowest taxonomic classification is shown for each ASVs, the letters denote: g (genus), o (order), f (family), sc (subclass), and c (class)

For the fungi libraries, we obtained 1,256,757 ITS reads after filtering and trimming, and we used them to evaluate the taxonomic composition. After the QIIME2 analysis, 213 ASVs were obtained of which 203 were retained for fungi classification. The results obtained with the UNITE database were too unspecific; therefore, we used BLAST to attempt a better classification. This analysis showed that the great majority of the fungal reads in cacao tissues belonged to the phylum Ascomycota (99.9%), and only a few reads belonged to the phyla *Basidiomycota* (0.02%) and *Mucoromycota* (0.01%). Within the *Ascomycota*, 99.7% of the reads belonged to the subphylum *Pezizomycotina* and few to the subphylum *Saccharomycotina* (0.3%) (Fig. 2).

Most genotypes and tissues had a similar fungal community (Fig. 2). The genus *Penicillium* was the most dominant, with 78.8% of reads (Fig. 2). Few samples had other fungi from the orders *Pleosporales* and *Hypocreales* and subclass *Chaetothyriomycetidae*. Some seedlings presented other dominant taxa than *Penicillium*. For instance, tissues from individuals 34 of AC9 and 18 of ICS95 had more abundance of fungi of the subclass *Chaetothyriomycetidae* (Fig. 2).

### Species Richness Analysis

The bacterial and fungal species richness indices were calculated for each sample to determine if there were significant differences between genotypes and tissue types (Tables S4, S5). The ITS species richness analysis did not show significant differences between genotypes or tissues (*p* > 0.05). The analysis of bacterial ASVs (with relative abundance > 1%) showed that the seedlings host a small number of species (on average, four ASVs per tissue). For some seedling tissues, we did not retrieve any bacterial reads. An average of six ASVs were found in the ITS libraries. The ICS95 rhizosphere libraries had a higher number of 16S rRNA ASVs (34 ASVs). To account for the phylogenetic distance among ASVs, we calculated the Faith phylogenetic diversity (PD) index [51]. There were significant differences between the diversity values of AC9 and the commercial genotypes (ICS95 and IMC67) as well as between AC9 and the recently liberated genotypes from AGROSAVIA (TCS01 and TCS19), no significant differences were observed when compared to other landraces. Similarly, the diversity values of ROS2 were significantly different to TCS19, IMC67, TCS01, and ICS95 (*p* < 0.05) (Table S4).

Regarding the tissues, the different diversity indices (Faith-PD and observed features) showed that the number of observed species and phylogenetic diversity was, on average, higher in roots than in the other tissues. Statistical evaluation of Faith-PD distances shows that there are significant differences (*p* < 0.05) between root tissue with cotyledon and leaf tissues (Table S5).

Interestingly, when the frequency of seedling emergence is mapped against the phylogenetic diversity index, the most diverse genotypes had a lower seedling emergence, except for ROS1, in which seed emergence was low (42.9%). For instance, the genotypes with the highest emergence values, TCS01 (71.0%), ROS2 (65.7%), and AC9 (62.9%), had the least diverse endophytic communities of bacteria, as reflected in Faith distance distributions. In contrast, IMC67 (51.4%) and TCS19 (51.6%), with lower emergence values, had more diverse communities. Similarly, ICS95 (40.7%) was the genotype with the most diverse bacterial community and had the lowest emergence values (Table S1). A significant negative correlation (Pearson's r) is observed between Faith PD and seed germination (*r* = -0.86, *p* < 0.05) and emergence (*r* = -0.82, *p* < 0.05) when the values for genotype ROS1 are excluded from the analysis.

### Bacterial and Fungal Composition of Different Genotypes and their Tissues

**Fig. 3:**
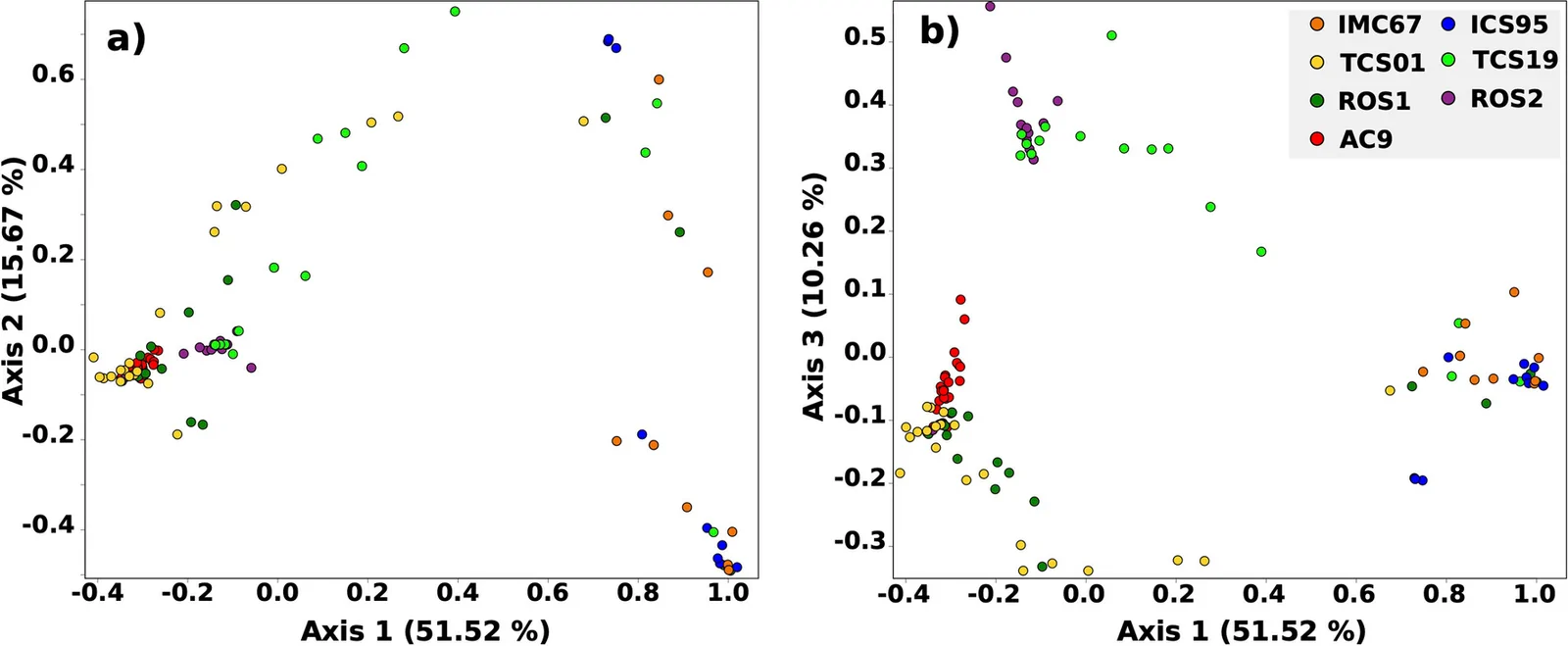
Principal Coordinate ordination Analysis of the seed-borne bacterial community present in different tissues of *T. cacao* genotypes. The IMC67 and ICS95 are the commercial genotypes, TCS01 and TCS19 are recently liberated genotypes from AGROSAVIA, and ROS1, ROS2, and AC9 are landraces. The PCoA was constructed using a weighted UniFrac distance matrix. **a** Axes 1 and 2 explain 67.2% of the variability. **b** Axes 1 and 3 explain 61.8%

A Principal Coordinate Ordination Analysis (PCoA) of the seed-borne bacteria was constructed with a matrix of weighted UniFrac distances between samples (Fig. 3). The PCoA was used to visualize the similarities or differences between the endophytic bacterial communities and to evaluate if the distribution was structured based on the genotype or plant tissues. The first, second, and third axes explained 51.5, 15.7, and 10.3% of the variability, respectively. When the samples were differentiated by genotype, three clustering groups were observed one composed of TCS01, AC9, and ROS1, a second of ROS2 and TCS19, and a third consisting primarily of IMC67 and ICS95 (Fig. 3). In contrast, when samples were analyzed by tissue, there was no clear clustering (data not shown).

We conducted a PERMANOVA analysis using the weighted and unweighted UniFrac distances to establish the significance of the genotype clustering (Tables S6, S8). Read subsampling was set to 500 reads because, at this depth, the species diversity was saturated based on the rarefaction curves. When analyzing the samples based on the weighted UniFrac distance, microbial communities were significantly different between genotypes except for commercial genotypes IMC67 and ICS95 (*p* < 0.05, Tables S6, S8). Interestingly, the weighted UniFrac average distance, having AC9 as a reference, showed a clear pattern where less domesticated genotypes had a shorter distance between them (AC9, ROS1, and ROS2), slightly higher distance with recently liberated ones (TCS01 and TCS19), and the most considerable distance with the commercial genotypes (IMC67 and ICS95, Fig. S2). Moreover, the comparison of plant tissue distances showed a significant difference between root and leaves, and between leaves and cotyledon (*p* < 0.05, Table S7). The comparison of tissue distances showed a significant difference between roots and other tissues indicating that the most differentiated endophytic community is in roots (*p* < 0.05, Table S9).

The Principal Coordinate Ordination Analysis (PCoA) of the fungi community was constructed with a matrix of weighted UniFrac distances and no clustering based on tissues or genotypes was observed (data not shown). Statistical analysis of the fungal community diversity between tissues showed no significant differences. Evaluation of genotypes showed few differences, the IMC67 genotype was significantly different from the others based on the unweighted UniFrac (*p* < 0.05, Table S10) and ROS1 based on the weighted UniFrac (*p* < 0.05) except with AC9 and ICS95 (Table S11).

### Isolation of Endophytic Bacteria and Evaluation of their Plant Growth-Promoting Capabilities

Bacteria endophytes were isolated from seeds and seedlings to determine their ability to promote plant growth. A total of 35 morphotypes were isolated from seedlings; 45.7% of the bacteria were isolated from the TCS01 genotype, 20.0% from the AC9, 11.4% from the TCS19, and 22.9% from the IMC67 genotype. Only two endophytic morphotypes were isolated directly from seeds of the AC9 genotype. We identified 33 of the isolated bacteria based on their 16S rRNA sequence. Most bacteria belonged to the *Pseudomonas* (42.4%) and *Bacillus* (30.3%) genera, while others belonged to *Stenotrophomonas* (12.1%), *Pantoea* (9.1%), and 6.1% to other genera (Table S12).

**Fig. 4:**
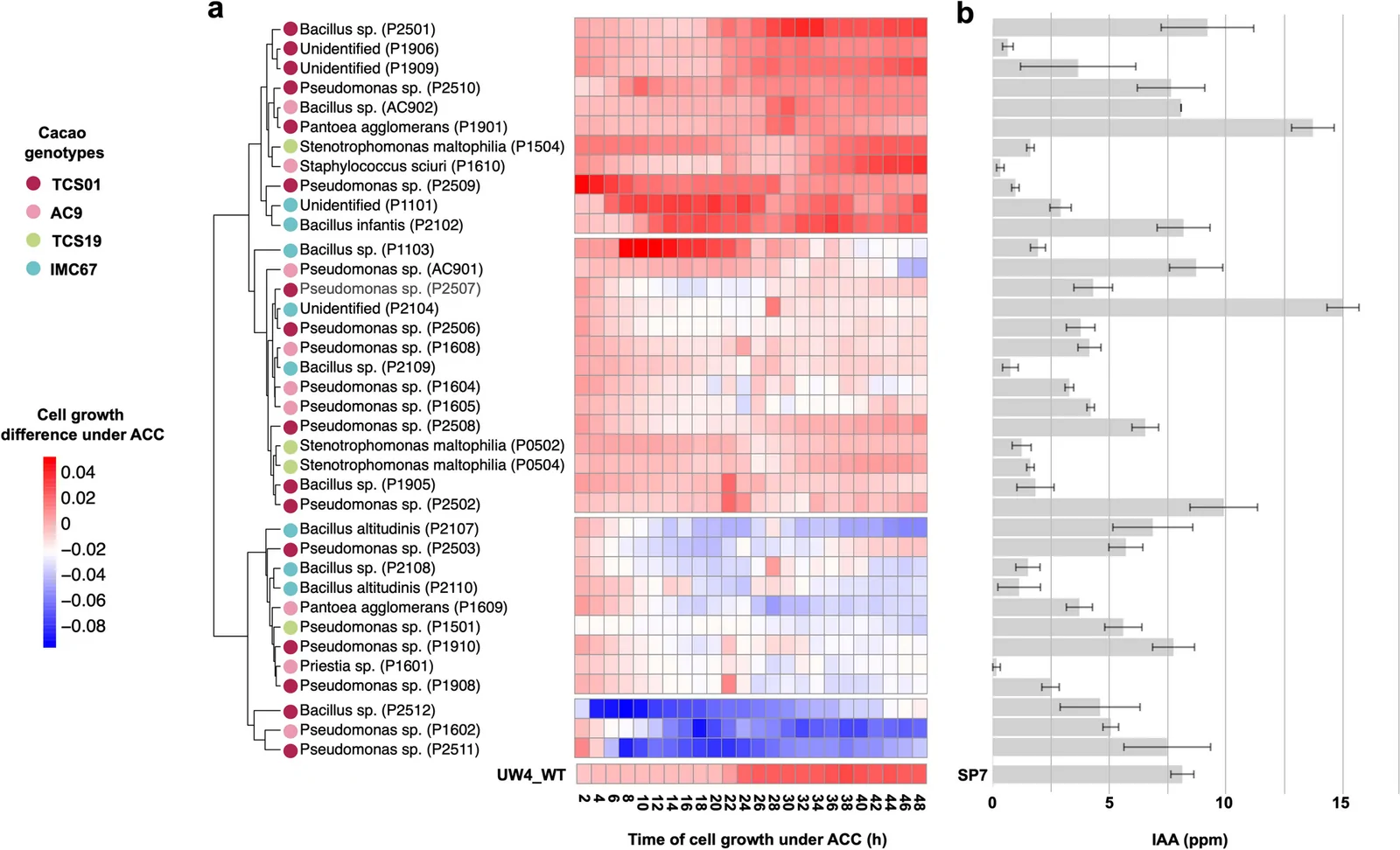
Evaluation of the plant growth-promoting capabilities of *T. cacao* seed-borne bacterial isolates. The ACC deaminase and indole acetic acid production (IAA) was evaluated for 37 bacterial isolates. **a** The heatmap represents the cell growth difference in a medium with ACC deaminase compared to a medium with MgSO_4_. Cell growth was monitored through absorbance (600 nm) every 2 h for 48 h. *Pseudomonas putida* UW4 WT was used as positive control for ACC deaminase production. The taxonomic classification based on 16S rRNA analysis is shown for each isolate, and the circles' colors show the isolate's genotype origin. The clades to the left of the heatmap cluster the isolates based on their cell growth profile. **b** The average IAA production and the standard deviations are shown for each isolate. *Azospirillum brasiliensis* SP7 was used as a positive control

The evaluation of the potential for plant growth promotion of the bacterial isolates was assessed to determine the 1-aminocyclopropane-1-carboxylic acid (ACC) deaminase activity and indole acetic acid (IAA) production. Twelve morphotypes (P2501, P1906, P1909, P2510, AC902, P1901, P1504, P1610, P2509, P1101, P2102, and P1103) were found to have ACC deaminase enzyme activity with average growth curves in ACC deaminase presence above the growth curve of the MgSO_4_ control treatment (Fig. 4a). A spectrophotometric calibration curve was done to estimate the IAA concentration. This calibration curve showed an *r*^*2*^ value of 0.99, indicating the method's reliability for estimating the production of IAA of the different morphotypes (Table S13). Among the evaluated morphotypes, *P. agglomerans* P1901, *Pseudomonas* sp. P2502 and strain P2104 (unidentified) produced more than 10.0 ppm of IAA, outperforming the control strain *Azospirillum brasiliensis* SP7, which generated 9.48 ppm after 72 h of growth (Fig. 4b). Furthermore, 11 isolates displayed IAA production levels exceeding 7.0 ppm after 72 h. These findings highlight the potential of these isolates to generate substantial amounts of IAA, suggesting their promising role in plant growth promotion.

## Discussion

Most studies on cacao endophytes are focused on vegetative tissues [13, 37] and only a few studies have addressed the diversity of seed-borne endophytes using culture-dependent approaches [52, 53]. Here, we explored vertically transmitted microbes in cacao by studying landraces and commercial cacao genotypes germinated under sterile conditions and we characterized these communities using amplicon sequencing.

The microbial diversity in cacao tissues showed that the roots (in all genotypes) presented the highest diversity and abundance of bacteria. Previous studies in cacao's roots [54], also recorded more bacteria in this tissue, and this pattern has also been observed in other crops, such as sugar cane [55]. This increased bacterial abundance in roots might be related to access to soil nutrients or abundant root exudate, since the root apical meristem usually has a significant concentration of exudates, providing energy and nutrient sources that promote bacterial cell division [55]. The second tissue with the highest bacteria abundance was the leaf tissue, which is to be expected, as they are among the most metabolically active tissues due to processes such as photosynthesis, evapotranspiration, and second metabolite excretion. In contrast, we found few reads associated with bacteria in seed tissues. One possible reason for this low abundance is that bacteria might be dormant or have little metabolic activity. For instance, a study on *Miscanthus* [56] indicated that endophytic bacteria in the seed are induced to become dormant and stay in low numbers. Once the seed matures and becomes more active upon imbibition, the endophytic bacteria multiply in the seedling tissue.

Using different cacao genotypes allowed us to assess the relationship between endophytic bacterial diversity (abundance and composition) and different domestication levels. Previous works have explored these patterns, finding contrasting results. For instance, some reports have shown that domestication reduces endophytic bacterial diversity in *Agave* plants [57], *Hordeum vulgare* [58], and *Malus domestica* [59], while in plants such as *Lactuca sativa*, *Triticum durum*, and *Phaseolus vulgaris* domestication has increased endophytic bacterial diversity [60, 61]. In our study, the tissues of commercial genotypes IMC67 and ICS95 had higher bacterial diversity than landraces. At first hand, the cacao pattern seems to fall into the increased diversity scenario; however, when normalized by mitochondria reads, the bacteria abundance is higher in landraces and exceptionally low in commercial genotypes. One possible explanation for this pattern is that crop management practices may affect the endophyte microbiome [4]. For instance, systemic fungicides can affect the endophytic community, which is vertically transmitted through seeds [62]. Plants of the IMC67 genotype, commonly used as rootstock, are treated with periodic applications of specific systemic and contact fungicides (*e.g.,* Copper Oxychloride and Ridomil), which would probably affect the composition and abundance of the microbiome. Another clear difference between commercial genotypes and landraces is the propagation mechanisms. In landraces, propagation occurs as a natural reproduction process. In contrast, in commercial genotypes, at least those used as clones, propagation occurs by grafting, which disrupts the natural process of plant reproduction, which might alter the transmission of endophytes to seeds and fruits.

The dynamics of microbial populations within plant tissues provide insights into the complex interplay of seed-borne endophytes. In the case of landraces and AGROSAVIA genotypes, most seedlings' tissues are dominated only by ASVs associated with *Pantoea* and *Pseudomonas* genera. These genera are commonly found in plant tissues and are known to coexist [63]. Some species of these genera are considered unharmful or beneficial endophytes, while others are reported as pathogenic. *Pantoea* is recognized as one of the main endophytic genera; it has been isolated from various tissues, such as maize seeds [64, 65] and wheat rhizosphere [66], and some of these isolates have shown to improve nutrient uptake, modulate growth, and stress-related phytohormones, among other benefits [63–65].

Similarly, *Pseudomonas* species can promote plant growth and provide biological control against fungal pathogens [67]. For example, *Ps. chlororaphi*s has been found to have antagonistic activity against *Phytophthora palmivora*, the causal agent of black pod disease [67, 68]. Our results indicate that either *Pantoea* or *Pseudomonas* dominate in almost all tissues, and their abundance may vary depending on ecological factors. Ecological competition may occur in cacao seed-borne tissues when one species is abundant, the other is not. In some cases, *P. agglomerans* can coexist with *Ps. syringae* [69] but compete with *Ps. savastanoi* for nutrients and space, inhibiting its growth through antibiosis activity [66].

Furthermore, our study found an inverse relationship between bacterial diversity and the emergence of cacao seedlings of different genotypes, where the higher abundance of *Pseudomonas* and *Pantoea* was associated with the highest seed emergence values, as observed for TCS01, ROS2, and AC9. Meanwhile, genotypes like IMC67, ROS1, and TCS19, with lower values of emergence success, had other taxonomic groups. Interestingly, ICS95, the genotype with the most diverse bacterial community, had the lowest emergence values. In this regard, Chesneau et al. [70] and Barret et al. [71] found a negative relationship between ASVs richness and seedling emergence and fitness in beans, reflected by a high proportion of non-germinated seeds and abnormal development of seedlings.

In contrast to the patterns observed for bacterial diversity, the ITS-5.8S amplicon study showed that the diversity of endophytic fungi in *T. cacao* was unaffected by the different domestication levels. The ITS-5.8S libraries for most seedling tissues were dominated by members of the phylum *Ascomycota*, compared to other phyla such as *Basidiomycota*. The presence of asexual morphs (hyphomycetous and coelomycetous) in *Ascomycota* fungi provides a good ability to colonize the internal tissues of plants, therefore they are reported more often as endophytes than other fungi taxa [72]. The genus *Penicillium* (order *Eurotiales*) was found in all plant tissues and was dominant in most *T. cacao* seedlings. Species of this fungal genus have been reported as endophytes of various plants, acting as growth promoters, improving disease resistance, and protecting against stress conditions [73, 74].

To elucidate the functional roles of the seed-borne bacterial communities, we isolated bacteria from the seedling tissues of the different evaluated genotypes. The characterization showed that most bacteria isolates have plant growth-promoting capabilities (67.6%). Most isolates corresponded to the *Bacillus* and *Pseudomonas* genera, consistent with the 16S rRNA amplicon characterization. Isolation of *B. subtilis* and *Pseudomonas* has been reported previously in cacao leaves [75], fruits, and seeds [53, 76]. Interestingly, most of our isolates display IAA production and ACC deaminase activity*.* We identified ten isolates that had an equivalent or higher IAA production and ACC deaminase activity than the reference strains; these were isolated principally from the TCS01 genotype (six isolates), which presented the highest germination of seeds (96.9%) and the highest emergence of seedlings (71.0%) under in vitro conditions. It has been reported that the ACC deaminase-producing bacterium improves seed germination and plant growth in different crops even under stress conditions [23, 25, 77, 78]. In turn, this also coincides with the fact that the TCS19 genotype presented the lowest germination percentage among those evaluated for bacterial isolates, and from which only one endophytic bacteria with this ACC deaminase activity was isolated. In cacao, the production of IAA and ACC deaminase by bacteria might stimulate the root hair and cotyledon cell expansion during seedling development and improve plant growth under stress conditions, as has been observed in other plants [23, 25, 77].

In addition, our approach for the normalization of bacteria relative abundance using organelle 16S rRNA reads facilitated the comparison of microbial abundance across tissues and genotypes. It provided consistent values among replicates and tissues of the same genotypes. Although a large proportion of 16S rRNA reads corresponded to organelle, the results showed that our method is suitable and practical for cases with low microbial complexity as saturation of species diversity was observed. There are alternative techniques that block the amplification of organelle 16S rRNA, thereby increasing the proportion of bacterial reads in the libraries, such as the Peptide Nucleic Acid—PNA primers [17, 79]. However, these techniques also prevent read normalization, which is a trade-off to consider.

## Conclusions

The investigation of seed-borne endophytes in cacao is relatively uncharted territory, with limited studies delving into their vertical transmission and establishment across various plant tissues during development. By exploring the diversity of these endophytes, we gain insights into the microbial communities established by vertical transmission. Our current study digs into this intriguing aspect, examining the composition of the seed-borne endophytic microbial community associated with *Theobroma cacao*. We further scrutinize the variability within these communities and the impact of domestication, drawing comparisons between widely used commercial, recently liberated genotypes, and landraces.

Our findings highlight a complex relationship between crop domestication and endophytic bacterial diversity, showing the intricate dynamics of microbial populations within plant tissues, particularly highlighting the dominance of *Pantoea* and *Pseudomonas* genera in less domesticated genotypes (landraces). These endophytes might play significant roles in growth modulation as we observed a better emergence of cacao seedlings where these two bacterial groups were dominant.

Our results also showed that commercial genotypes had a reduced bacteria abundance, probably influenced by agricultural practices and propagation mechanisms, paving the way for colonizing a more diverse range of taxonomic groups. These observations underscore the importance of incorporating abundance quantification methods, such as qPCR or read normalization using mitochondrial or chloroplast 16S rRNA copies, to fully understand the effect of agricultural practices and domestication on the plant microbiome.

On the other hand, the endophytic fungal diversity in *T. cacao* seedlings is predominantly composed of Ascomycota members, with the genus *Penicillium* being ubiquitous across all plant tissues. Further studies would be necessary to establish if these fungi might play crucial roles in growth promotion or increasing resistance against pathogens, holding promise as biocontrol agents.

In conclusion, our study illuminates the intricate interplay between domestication, crop management, and microbiome diversity. These insights are paramount to agriculture, as they elucidate the profound impact of human-driven practices on crop microbiomes. This knowledge could potentially inform strategies to enhance plant health, productivity, propagation alternatives, and resilience against diseases.

## Supplementary Information

Below is the link to the electronic supplementary material.

Supplementary Fig. S1 (JPG 743 KB) 16S rRNA and ITS-5.8S libraries generated in each of the plant tissues of the T. cacao genotypes. a) Root, b) Stem, c) Leaf, d) Cotyledon.

Supplementary Fig. S2 (JPG 498 KB) Distribution of the weighted UniFrac distance between AC9 and the other T. cacao genotypes. The PERMANOVA analyses supported significant differences (*p* < 0.05) between AC9 and the other genotypes. However, the difference increases with domestication; the distance is smaller between AC9 and recently liberated genotypes from AGROSAVIA (TCS01, TCS09) and landraces (ROS1, ROS2), and larger between AC9 and commercial genotypes (IMC67, ICS95). 

Supplementary file3 (XLSX 74 KB)

## Data Availability

The bacterial and fungal sequence data generated in this study using MiSeq have been deposited and are available in the NCBI Sequence Read Archive (SRA) under BioProject PRJNA1011283.
