# Supplementary figures and images for: Implications of Domestication in Theobroma cacao L. Seed-Borne Microbial Endophytes Diversity
Source: Microb Ecol. 2024 Aug 28;87(1):108. doi: 10.1007/s00248-024-02409-9 (PMC11358227; doi:10.1007/s00248-024-02409-9)

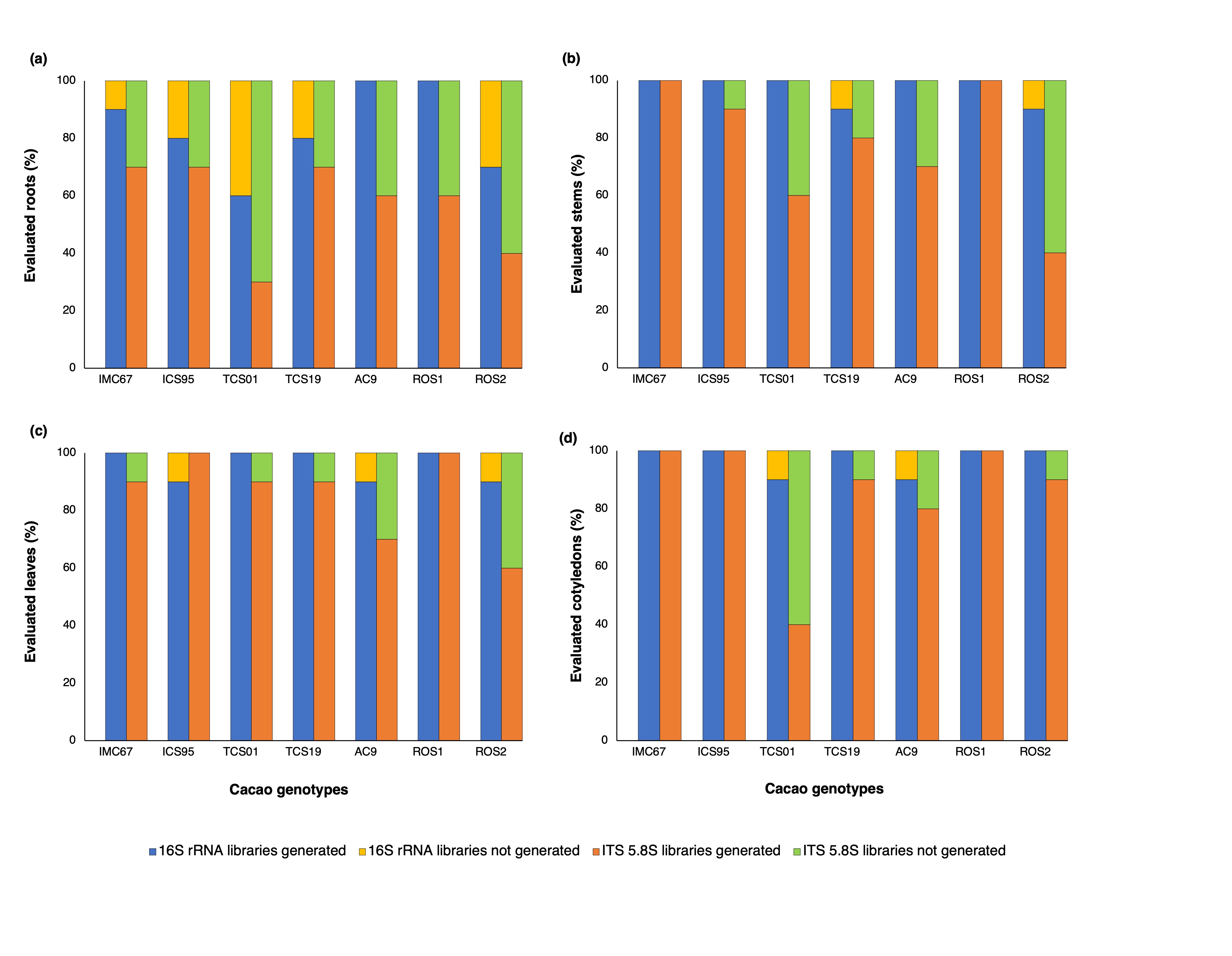

Supplement: Supplementary file 1 — Supplementary Fig. S1 (JPG 743 KB) 16S rRNA and ITS-5.8S libraries generated in each of the plant tissues of the T. cacao genotypes. a) Root, b) Stem, c) Leaf, d) Cotyledon. [file 248_2024_2409_MOESM1_ESM.jpg]

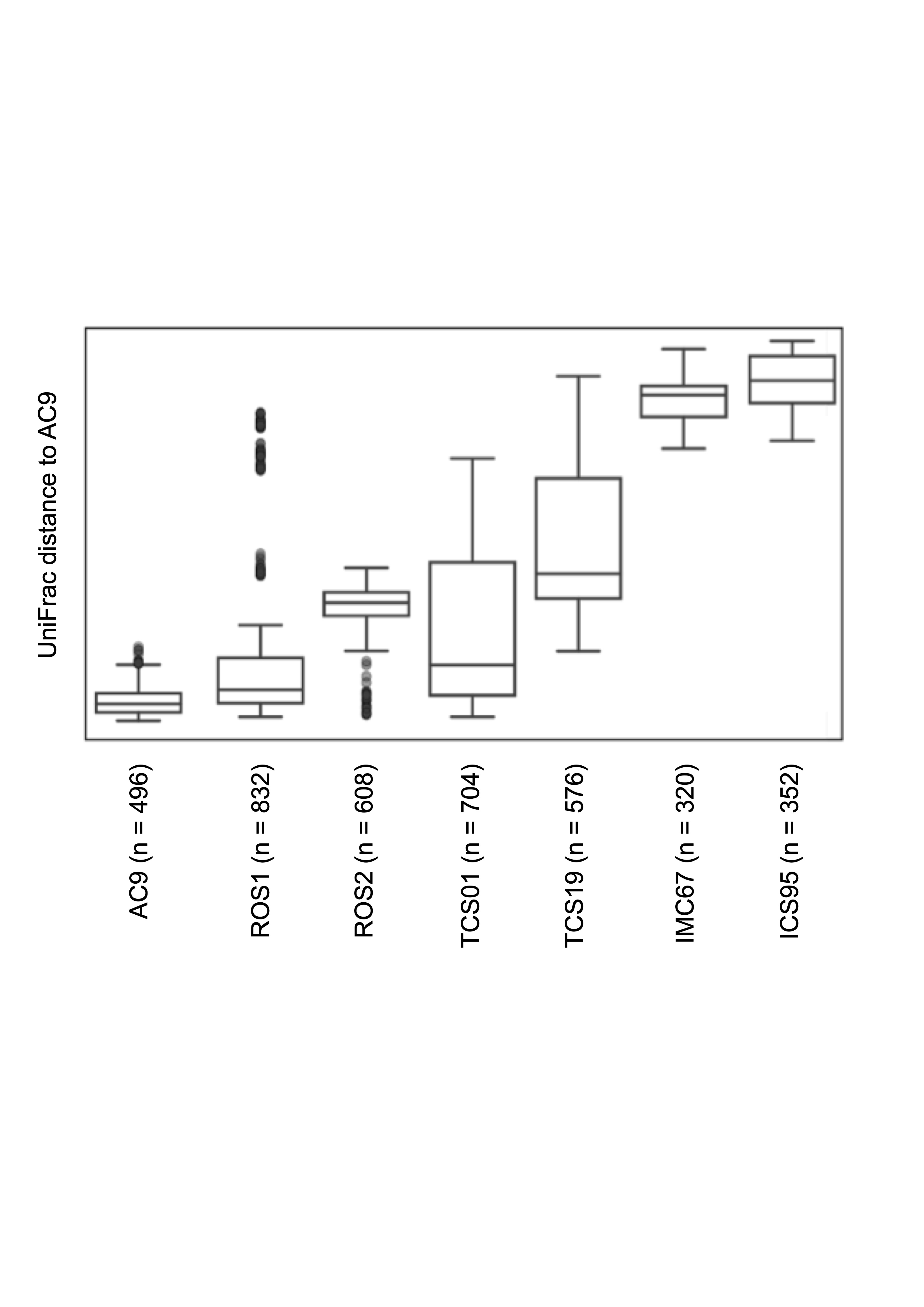

Supplement: Supplementary file 2 — Supplementary Fig. S2 (JPG 498 KB) Distribution of the weighted UniFrac distance between AC9 and the other T. cacao genotypes. The PERMANOVA analyses supported significant differences (p < 0.05) between AC9 and the other genotypes. However, the difference increases with domestication; the distance is smaller between AC9 and recently liberated genotypes from AGROSAVIA (TCS01, TCS09) and landraces (ROS1, ROS2), and larger between AC9 and commercial genotypes (IMC67, ICS95). [file 248_2024_2409_MOESM2_ESM.jpg]
